# Postmastectomy Reconstruction in Male Breast Cancer

**DOI:** 10.1155/2022/5482261

**Published:** 2022-03-29

**Authors:** Romina Deldar, Adaah A. Sayyed, Parhom Towfighi, Nathan Aminpour, Olutayo Sogunro, Jennifer D. Son, Kenneth L. Fan, David H. Song

**Affiliations:** ^1^Department of Plastic & Reconstructive Surgery, MedStar Georgetown University Hospital, Washington, DC, USA; ^2^Georgetown University School of Medicine, Washington, DC, USA; ^3^Department of General Surgery, MedStar Georgetown University Hospital, Washington, DC, USA

## Abstract

**Introduction:**

Less than 1% of all breast cancers are diagnosed in males. In females, postmastectomy breast reconstruction is associated with increased patient satisfaction. However, there is a paucity of literature describing reconstructive options for postmastectomy deformity in the male chest. The purpose of this systematic review was to evaluate postmastectomy reconstruction outcomes in males with breast cancer.

**Methods:**

A systematic review was performed in accordance with PRISMA guidelines. Ovid MEDLINE, Embase, Cochrane, and Web of Science were queried for records pertaining to the study question using medical subject heading (MeSH) terms such as “male breast cancer,” “mastectomy,” and “reconstruction.” No limitations were placed on the year of publication, country of origin, or study size. Study characteristics and patient demographics were collected. Primary outcomes of interest included postoperative complications, recurrence rate, and mortality rate.

**Results:**

A total of 11 articles examining 29 male patients with breast cancer who underwent postmastectomy reconstruction were included for analysis. Literature was most commonly available in the form of case reports. The average age was 59.6 +/−11.4 years. Reconstruction methods included fat grafting (*n* = 1, 3.4%), silicone implants (*n* = 1, 3.4%), and autologous chest wall reconstruction with local flaps (*n* = 26, 89.7%). Postoperative complications occurred in two patients (6.8%), including partial nipple necrosis (*n* = 1) and hypertrophic scarring (*n* = 1). Of the studies reporting patient satisfaction, all patients were pleased with the aesthetic appearance of their chest.

**Conclusion:**

This systematic review revealed the limited availability of research regarding postmastectomy chest reconstruction in males with breast cancer. Nevertheless, the evidence available suggests that reconstruction can restore a patient's body image and, thus, should be regularly considered and discussed with male patients. Larger studies are warranted to further shed light on this population.

## 1. Introduction

Less than 1% of all breast cancers are diagnosed in males; however, the incidence is rising [1–3]. According to the American Cancer Society, about 2,710 new cases of invasive breast cancer will be diagnosed in the United States in 2022 [4]. Less than 0.2% of cancer-related deaths in men can be attributed to male breast cancer [4]. The majority of male breast cancers are invasive ductal carcinoma, accounting for up to 90% of cases [5]. Estimates of in situ carcinoma in men are approximately 10% [4, 6]. Invasive lobular carcinoma is rare in men due to a lack of terminal breast lobules [7]. Men with breast cancer are significantly more likely to have hormone-receptor positive tumors, nodal metastases, and to be diagnosed at a more advanced stage than their female counterparts [1, 8]. Tumors are typically in the central subareolar location of the male breast and often involve the nipple [9].

Several risk factors have been identified for male breast cancer. Underlying genetic alterations differ between male and female breast cancers. Common genetic mutations in male breast cancer occur in the BRCA-2 gene, CHEK2 gene, and PALB2 gene [4, 10, 11]. Klinefelter's syndrome, characterized by an XXY genotype, is associated with testicular dysgenesis, gynecomastia, and an altered balance of androgens and estrogens, which confers a 50-fold increased risk of developing male breast cancer [3, 4]. Exogenous causes of hyperestrogenism in males, such as estrogen treatment in prostate cancer or hormone therapy for male-to-female transgender individuals, can increase the risk of male breast cancer [12, 13]. Endogenous causes of higher estrogen levels including obesity, cirrhosis, mumps orchitis, undescended testes, or testicular injury have also been associated with a higher risk of male breast cancer [3, 14]. Since breast cancer is traditionally regarded as a “female disease,” a diagnosis of breast cancer may induce feelings of demasculinization, altered body image, and embarrassment in males [15, 16]. In a survey of 28 males, 43% reported that a diagnosis of breast cancer may cause them to question their masculinity [9].

Current treatment for male breast cancer has largely been extrapolated from female breast cancer, despite the unique anatomy of the male breast. Due to the paucity of male breast tissue and the typical central subareolar tumor location, the standard treatment in male breast cancer is a modified radical mastectomy, which includes removal of the nipple and axillary node sampling or dissection [6, 8, 17, 18]. This results in an asymmetrical chest, which can create a substantial emotional burden and negative self-image in males [8, 18]. Postmastectomy breast reconstruction has been found to increase patient satisfaction and psychosocial well-being in female patients [19–21]. Although postmastectomy reconstruction options for women have been well described, there is a paucity of literature describing reconstructive options for postmastectomy deformity in the male chest. Oftentimes, postmastectomy reconstruction is not discussed or offered to men during their preoperative consultation, despite federal law requiring health plans that pay for mastectomy to also cover breast reconstruction in both men and women [22]. The purpose of this systematic review was to evaluate postmastectomy reconstruction in males with breast cancer.

## 2. Methods

This systematic review adhered to the Preferred Reporting Items for Systematic Reviews and Meta-Analyses (PRISMA) guidelines [23] and the Cochrane handbook of systematic reviews [24]. A systematic search of Ovid MEDLINE, Embase, Cochrane, and Web of Science was performed using medical subject heading (MeSH) terms and keywords including but not limited to “male breast cancer,” “mastectomy,” and “reconstruction”.

### 2.1. Study Selection

**Figure 1:**
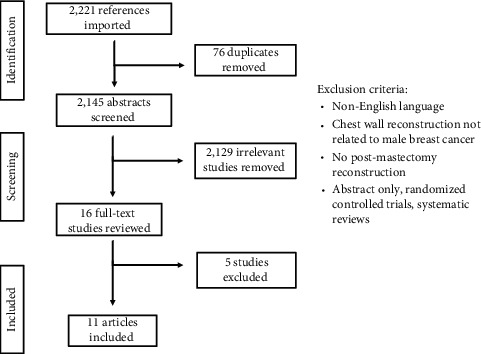
Study selection process flowchart.

Using the Rayyan (Qatar Computing Research Institute, Doha, Qatar) systematic review web application, two independent reviewers (P.T. and N.A.) screened each citation. First, studies were screened for relevance based on titles and abstracts. In the event that a screening decision was not unanimous, a third reviewer (R.D.) was consulted to discuss their reasoning until consensus was reached. The remaining studies then underwent full-text review. For inclusion in this study, all papers met the following criteria: (1) addressed males with breast cancer who underwent resection surgery and (2) reconstruction with fat grafting/lipofilling, autologous reconstruction, or implant-based reconstruction. Due to the scarce amount of literature on this topic, no restrictions were set on year of publication, country of origin, or study size. Studies were excluded if they did not report on males with breast cancer, postmastectomy reconstruction, or were not written in the English language. A flowchart outlining the study selection process is shown in Figure 1. Each study was analyzed for patient demographics, breast cancer characteristics, type of mastectomy and reconstruction, postoperative complications, recurrence and mortality rates, and follow-up period.

## 3. Results

The majority of articles were case reports (*n* = 9, 81.8%), followed by retrospective cohort studies (*n* = 2, 18.2%). Included articles described a total of 29 male patients with breast cancer who underwent postmastectomy reconstruction.

### 3.1. Patient Demographics and Cancer Characteristics

General study characteristics and breast cancer characteristics of the patients in each study are described in Table 1. Of the 10 studies that explicitly stated patients' age at the time of reconstruction, the average age was 59.6 ± 11.4 years. Only one study reported the body mass index (BMI) in its two patients (35 and 59 kg/m^2^) [33]. Invasive ductal carcinoma was the most common the breast cancer type (*n* = 27, 93.1%), followed by papillary ductal carcinoma (*n* = 1, 3.4%). One study [27] did not specify breast cancer type. All tumors were hormone-receptor positive among the studies that reported receptor status (*n* = 6). One patient underwent neoadjuvant chemotherapy to decrease tumor size prior to mastectomy [32], and two patients underwent adjuvant chemotherapy with hormone therapy [31, 33].

### 3.2. Mastectomy and Chest Reconstruction

Among the 29 patients included in this review, two patients (6.8%) underwent bilateral mastectomy [26, 33]. Ten patients (34.5%) underwent radical mastectomy and five patients (17.2%) underwent modified radical mastectomy. Four studies [1, 25–27] did not identify the type of mastectomy performed, and one study [29] did not specify mastectomy technique among the patients who underwent reconstruction.

Seven studies reported immediate reconstruction following mastectomy. Reconstruction methods included fat grafting [25] (*n* = 1; 3.4%), silicone implants [26] (*n* = 1; 3.4%), and autologous chest wall reconstruction with local flaps [1, 27–29, 31–34] (*n* = 26; 89.7%). One patient (3.4%) underwent primary closure of the mastectomy incision followed by delayed nipple-areolar complex (NAC) reconstruction [30]. Of the 26 local flaps described, myocutaneous latissimus dorsi (LD) flaps (*n* = 9; 34.6%) were performed most commonly, followed by transverse rectus abdominis muscle (TRAM) flaps (*n* = 5; 19.2%). Other local flaps included thoracoepigastric flap, deltopectoral flap, internal-external oblique flap, and cutaneous flaps (Table 2).

NAC reconstruction occurred in three patients. Two case reports described using a local skate flap with a skin graft [25, 34], and another utilized a full-thickness skin graft and a subdermal local flap to recreate the areola and nipple, respectively [30]. There were no reports of nipple tattooing.

### 3.3. Complications, Long-Term Outcomes, and Patient Satisfaction

Of the eight studies that commented on postoperative complications, six articles reported no complications during the recovery process. One study described partial nipple necrosis that did not require revision [25], and another study reported hypertrophic scarring in one of its two patients, which was treated with steroids [33].

Follow-up duration was mentioned in nine of the 11 studies; Bamba et al. [26] did not mention follow-up in their case report, and another study [29] provided a median follow-up of 58 months among their entire cohort of males with breast cancer, not just those who underwent postmastectomy reconstruction. Thus, in the nine studies that mentioned follow-up for our target population, the average follow-up was 27.9 +/−19.6 months. Di Benedetto et al. described a mortality rate of 45.5% (five of 11 patients who underwent postmastectomy reconstruction) [28]. Seven case reports stated that eight of their patients were alive at most recent follow-up. One case report highlighted that its patient experienced three local recurrences, occurring at six, nine, and 18 months [31]. Three studies [26, 27, 29] did not report recurrence, mortality, or follow-up specifically for patients who underwent reconstruction.

Five studies [25, 26, 30, 33, 34] commented on patient satisfaction following reconstruction, in which all patients were reported to be pleased with the appearance of their chest postreconstruction.  Table 3 summarizes complications, long-term outcomes, and patient satisfaction in males who underwent postmastectomy reconstruction.

## 4. Discussion

The primary aim of this review was to systematically assess the few available studies on postmastectomy reconstructive methods in males with breast cancer. In general, the literature on this subject is scarce, and there is a lack of high-quality study designs. Only two of the studies included in this review were retrospective cohort studies, while the remainder were case reports.

Local flaps were the most common reconstruction method performed following mastectomy in males. LD flaps from the back were performed most commonly, followed by the TRAM flap. Autologous reconstruction to restore the male chest contour necessitates important aesthetic considerations, such as hair pattern and thickness of subcutaneous fat and skin. The myocutaneous LD flap is an appealing option due to its reliability, proximity to the mastectomy defect, simplicity of use, and similarity in thickness of subcutaneous fat and skin provided [34]. However, in men with hairless backs, an LD flap will create a noticeable unaesthetic patch rather than restoring normal chest cosmesis. This was the case reported by Spear et al. [34], who instead performed a TRAM flap, as the patient's abdomen demonstrated a similar hair pattern and thickness to the patient's chest. The choice of local flap performed should be based on the patient's motivations for pursuing postmastectomy reconstruction.

Only one article reported the use of fat grafting to fill in the residual chest contour defect following mastectomy [25]. Fat grafting is a straightforward technique associated with low complications and minimal donor site morbidity [35]. Reconstruction of the male breast may require small graft volumes and fewer operative sessions to provide a natural feel and satisfactory cosmesis [25]. However, this technique is not without challenges, which include the varying “take” of fat grafting and the potential for fat necrosis and subsequent infection to occur [36]. Additionally, the theoretical risk of local cancer recurrence in fat-grafted breasts secondary to malignant transformation of transferred adipocytes and adipose-derived stem cells exists [37, 38] but has not been observed in multiple studies [37, 39].

Eight of the 11 studies mentioned complication rates, of which only two complications were reported. Al-Kalla et al. reported partial necrosis of the reconstructed nipple in their patient; however, this did not require revision [25]. One of the two patients in the study by Schaverien et al. experienced hypertrophic scarring, which was treated with a local steroid injection [33]. However, the included studies varied greatly in follow-up duration (ranging from six months [25] to three years [28]) and three studies did not mention follow-up at all. This is particularly relevant when considering complications unique to breast reconstruction, such as capsular contracture, which may occur years after implant placement [40, 41].

Despite a federal law mandating that health care plans cover postmastectomy reconstruction in men and women, surgeons do not regularly offer male patients this option [22]. This is likely due to the surgeon's assumption that males are not concerned by the appearance of their chest following breast cancer surgery [33]. However, this generalization can be detrimental and lead to negative psychosocial impacts for men with breast cancer. The patient in the case report by Spear et al. pursued local flap reconstruction 16 months after his mastectomy because he had become depressed about his physical appearance and did not feel comfortable participating in outdoor activities where his chest might be visible [34].

Besides having to cope with a predominantly female disease, men with breast cancer also deal with the physical changes of their chest after surgery. Some men may develop a negative body image as men often associate their chest with masculinity [42]. Many of the articles included in this review reported patient satisfaction following the reconstruction of their chests. Similar to what has been reported in female patients [21], males with breast cancer who undergo postmastectomy chest wall reconstruction may experience a strong psychological benefit.

There were several limitations in this study. Because of the rarity of male breast cancer, the literature lacks high-quality reports that detail postmastectomy reconstruction. Thus, there were only a small number of articles, mostly case reports, available for this review. Secondly, the follow-up duration varied among the studies, which may have affected the occurrence of complications reported. Indications for reconstruction type were not always reported. Finally, we cannot conclude whether the outcomes we observed resulted from patient disease severity or surgical procedure selection. Despite these limitations, the evidence available suggests that postmastectomy chest reconstruction in males can be beneficial from an aesthetic and psychosocial aspect and should be offered regularly in the preoperative setting.

## 5. Conclusion

This systematic review revealed that there is limited research specific to chest wall reconstruction following breast cancer resection in males. Postmastectomy chest reconstruction should be regularly considered and discussed with men who have breast cancer. Larger studies are warranted to further shed light on this patient population.

## Figures and Tables

**Table 1:** Patient demographics and cancer characteristics.

Author, year	Study type	Country	Patients (n)^a^	Age (yrs)	Breast CA type; stage	Tumor size	Hormone receptor	Medical therapy
Al-Kalla and Komorowska-Timek, 2014 [25]	Case report	USA	1	68	IDC; stage 1	1 × 2 cm	ER^+^/PR^+^	Not reported
Bamba et al., 2017 [26]	Case report	USA	1	78	IDC	Not reported	Not reported	Not reported
Banys-Paluchowski et al., 2016 [1]	Case report	Germany	1	62	Papillary CA; stage 2	16 cm diameter	ER^+^/PR^+^	Not reported
Danino et al., 1998 [27]	Case report	France	1	57	Stage 4	Not reported	Not reported	Not reported
Di Benedetto et al., 1997 [28]	Retrospective cohort	Italy	11	64 + 6.7	IDC; early: 2 intermediate: 6 advanced: 2	Not reported	Not reported	Not reported
Elshafiey et al., 2011 [29]	Retrospective cohort	Egypt	8 (25%)	Not specified^b^	IDC	Not reported	Not specified^b^	Not reported
Giunta et al., 2017 [30]	Case report	Italy	1	46	IDC; stage 1	Not reported	ER^+^/PR^+^	None
Igun, 2000 [31]	Case report	Nigeria	1	35	IDC; stage 3	3 × 3 cm	Not reported	Adjuvant tamoxifen, chemotherapy
Nakao et al., 2002 [32]	Case report	Japan	1	59	IDC; stage 4	9 × 6 cm	Not reported	Neoadjuvant chemotherapy
Schaverien et al., 2013 [33]	Case report	Scotland	2	35, 59	IDC; stage 2, stage 1	3 × 2 cm 1 × 1 cm	ER^+^/PR^−^ER^+^/PR+	Adjuvant tamoxifen, chemotherapy, XRT
Spear et al., 1997 [34]	Case report	USA	1	49	IDC, stage 2	2.2 cm	ER^+^/PR^+^	None

Age is presented as *n* or mean ± standard deviation. ^a^Number of patients who underwent postmastectomy reconstruction. ^b^Not specified for the 8 patients who underwent postmastectomy reconstruction. CA: carcinoma; IDC: invasive ductal carcinoma; MRM: modified radical mastectomy; XRT: radiation therapy.

**Table 2:** Mastectomy and reconstruction characteristics.

Author, year	Mastectomy type	Chest reconstruction	Type of local flap	Timing of reconstruction	NAC reconstruction
Al-Kalla and Komorowska-Timek, 2014 [25]	Not specified	Fat grafting	N/A	Immediate	Skate flap with skin graft
Bamba et al., 2017 [26]	Bilateral, not specified	Silicone implants	N/A	Delayed	None
Banys-Paluchowski et al., 2016 [1]	Not specified	Local flap	Pedicled LD myocutaneous flap	Immediate	None
Danino et al., 1998 [27]	Not specified	Local flap	Rotational internal-external oblique flap	Delayed	None
Di Benedetto et al., 1997 [28]	MRM: 2 radical mastectomy: 9	Local flap (*n* = 11)	Thoracic fasciocutaneous flap: 2 thoracoepigastric flap: 2 LD myocutaneous flap: 5 TRAM flap: 2	Immediate	None
Elshafiey et al., 2011 [29]	Not specified^a^	Local flap (*n* = 8)	LD myocutaneous flap: 3 TRAM flap: 1 cutaneous local flap: 4	Immediate	None
Giunta et al., 2017 [30]	MRM	Primary closure with NAC reconstruction	N/A	Delayed NAC reconstruction	FTSG for areola, local flap for nipple
Igun, 2000 [31]	MRM	Local flap	TRAM flap	Immediate	None
Nakao et al., 2002 [32]	Radical mastectomy	Local flap	Pedicled deltopectoral flap	Immediate	None
Schaverien et al., 2013 [33]	Bilateral simple mastectomy	Local flap	Rotational hatchet flaps	Immediate	None
Spear and Bowen, 1997 [34]	MRM	Local flap	TRAM flap	Delayed	Skate flap with FTSG to recreate areola

^a^Not specified for the 8 patients who underwent postmastectomy reconstruction. FTSG: full-thickness skin graft; LD: latissimus dorsi; MRM: modified radical mastectomy; TRAM: transverse rectus abdominis muscle; NAC: nipple-areola complex.

**Table 3:** Postoperative complications and long-term outcomes.

Author, year	Complications	Patient satisfaction	Recurrence	Mortality	Follow-up duration
Al-Kalla and Komorowska-Timek, 2014 [25]	Partial necrosis of nipple	Satisfied	0	0	6 months
Bamba et al., 2017 [26]	None	Satisfied	Not reported	Not reported	Not reported
Banys-Paluchowski et al., 2016 [1]	None	Not reported	0	0	24 months
Danino et al., 1998 [27]	Not reported	Not reported	Not reported	Not reported	Not reported
Di Benedetto et al., 1997 [28]	Not reported	Not reported	Not reported	5 (45.5%)	36 ± 22.1 months
Elshafiey et al., 2011 [29]	Not specified^a^	Not reported	Not specified^a^	Not specified^a^	Not specified^a^
Giunta et al., 2017 [30]	None	Satisfied	0	0	18 months
Igun, 2000 [31]	None	Not reported	Local × 3	0	24 months
Nakao et al., 2002 [32]	None	Not reported	0	0	24 months
Schaverien et al., 2013 [33]	Hypertrophic scarring	Satisfied	0	0	10 months; 17 months
Spear and Bowen, 1997 [34]	None	Satisfied	0	0	12 months

^a^Not specified for the 8 patients who underwent postmastectomy reconstruction.

## Data Availability

The data that support the findings of this study are available on request from the corresponding author.
